# Evolution and Phylogeny of Soybean Mosaic Virus Based on 143 Complete Genomes

**DOI:** 10.3390/ijms24010022

**Published:** 2022-12-20

**Authors:** Hoseong Choi, Yeonhwa Jo, Soo Yeon Choi, Sang-Min Kim, Yu Mi Choi, Jin-Sung Hong, Bong Choon Lee, Won Kyong Cho

**Affiliations:** 1 Plant Genomics and Breeding Institute, Seoul National University, Seoul 08826, Republic of Korea; 2 College of Biotechnology and Bioengineering, Sungkyunkwan University, Suwon 16419, Republic of Korea; 3 Crop Foundation Division, National Institute of Crop Science, Rural Development Administration, Wanju 55365, Republic of Korea; 4 Agrobiodiversity Center, National Institute of Agricultural Sciences, Rural Development Administration, Jeonju 54874, Republic of Korea; 5 Department of Applied Biology, Kangwon National University, Chuncheon 24341, Republic of Korea

**Keywords:** soybean mosaic virus, genome, evolution, phylogeny, diversity

## Abstract

*Soybean mosaic virus* (SMV) of the genus *Potyvirus* is an important virus in cultivated soybeans. Here, we obtained 7 SMV genomes from soybean germplasms using RNA sequencing and conducted a comprehensive evolutionary and phylogenetic study of 143 SMV genomes derived from 10 plant species and 12 countries. The phylogenetic tree we constructed using coding DNA sequences revealed the existence of nine clades of SMV isolates/strains. Recombination analysis revealed 76 recombinant events and 141 recombinants in total. Clades 1 and 3 contain the most common SMV pathotypes, including G1 through G7, which are distributed worldwide. Clade 2 includes several Chinese SMV pathotypes. The SMV isolates were further divided into two groups. The SMV isolates in the first group, including clades 8 and 9, were identified from *Pinellia* and *Atractylodes* species, whereas those in the second group (clades 1 through 7) were mostly found in cultivated soybeans. The SMV polyprotein undergoes positive selection, whereas most mature proteins, except for the P1 protein, undergo negative selection. The P1 protein of SMV isolates in group 1 may be highly correlated with host adaptation. This study provides strong evidence that recombination and plant hosts are powerful forces driving the genetic diversity of the SMV genome.

## 1. Introduction

*Soybean mosaic virus* (SMV) is a member of the genus *Potyvirus* in the family *Potyviridae* [1]. The main hosts of SMV are cultivated (*Glycine max* (L.) Merr.) and wild soybeans (*Glycine soya* Sieb. & Zucc.). SMV infects several plant species in the family Fabaceae [2]. In soybean plants, SMV infection results in foliar viral disease symptoms such as distorted and wrinkled leaves with mosaic or mottled patterns of color patches. In addition, SMV infection causes a reduction in seed size and the mottling of seed coats [3]. Moreover, dwarfism and a reduction in seed size and pod number were observed in soybean plants infected with SMV. Because it reduces the quality and quantity of soybean seed production, SMV is currently regarded as one of the most devastating viruses of cultivated soybeans [4]. 

SMV is usually transmitted by diverse aphids in a non-persistent manner [5]. It is also transmitted through seeds, with a seed transmission rate of up to 5% [6]. Therefore, spraying insecticides or planting virus-free seeds is useful for preventing the spread of SMV during soybean cultivation. 

Several research groups have studied the interaction between SMV and soybeans by identifying and characterizing the resistance (*R*) genes against SMV and proteins that interact with known SMV strains [1]. As a result, many soybean cultivars that are resistant to diverse SMV strains with different pathogenicities have been identified. Of the known SMV resistance loci, the *Rsv1* locus that confers resistance to seven SMV strains (G1 through G7) was first identified in the PI96983 cultivar [7]. The dominant *Rsv1* gene, which is located on chromosome 13, is highly complex. For example, a previous study identified a cluster of *R* gene candidates composed of several NB-LRR (nucleotide-binding leucine-rich repeat) family genes [8].

The SMV genome consists of single-stranded positive-sense RNA. It is approximately 9000 nucleotides (nt) in length with a poly(A) tail at the 3′ end of its genome. The SMV genome has two open reading frames (ORFs), which encode a polyprotein and PIPO (pretty interesting *Potyviridae* ORF). The polyprotein precursor is processed into 10 functional proteins, namely, protein 1 (P1), helper component protease (HC-Pro), protein 3 (P3), 6 kDa protein 1 (6K1), cylindrical inclusion body (CI), 6K2, nuclear inclusion protein a-viral genome-linked protein (NIa-VPg), NIa-Pro, nuclear inclusion protein b (NIb), and coat protein (CP) by the cellular proteolytic activity of three viral-encoded proteinases. 

Since the complete genome sequencing of the SMV strains G2 and G7 [9,10,11], many complete SMV genome sequences have been reported in diverse countries, including South Korea, China, the United States of America (USA), and Iran [12,13,14,15]. Furthermore, several high-throughput sequencing (HTS) technologies have facilitated the complete genome sequencing of known and novel viruses that infect a wide range of plant species [16].

Here, we obtained complete genome sequences for seven SMV isolates from soybean germplasms. After combining all available SMV genome sequences, we performed comprehensive analyses to elucidate the molecular evolution and phylogenetic relationships of the known SMV isolates/strains.

## 2. Results

### 2.1. Complete Genome Sequences of Seven SMV Isolates from Soybean Germplasms Obtained via RNA Sequencing

We examined viral infections in soybean germplasms using RT-PCR. Of these, we selected 10 soybean germplasms that were infected with SMV for RNA sequencing. Bioinformatic analyses identified 49 SMV-associated contigs ranging from 202 to 9592 bp in 4 of the 10 libraries. In addition, we obtained seven complete SMV genomes from four libraries, referred to as GR1, GR2, GR3, and GR4. In the GR1 library, two SMV isolates were identified. These isolates were referred to as GR1-1 and GR1-2, and both had genomes that were 9569 nucleotides (nt) long. We also obtained the complete genome sequences of three SMV isolates from the GR2 library. These isolates were referred to as GR2-1, GR2-2, and GR2-3, and all of them had genomes that were 9571 nt in length. We identified only single SMV isolates in the G3 and G4 libraries. These isolates were referred to as GR3 (9571 nt) and GR4 (9566 nt), respectively.

The SMV genome contains two ORFs and contains a poly (A) tail (Table 1). In the case of the SMV isolate GR1-1, ORF1 (positions 117 to 3066) encodes a polyprotein, whereas ORF2 (positions 2867 to 3094) encodes a PIPO protein. The BLASTN results showed that six SMV isolates (G1-1 and G1-2) had sequence similarity to the known SMV isolate GYGI-p (GenBank MT603834.1) identified from soybeans in Gyeonggi Province, Korea, with 99% coverage and 97.14% nucleotide identity. In addition, the SMV strain GR4 showed sequence similarity to SMV strain G5 (GenBank FJ640980.1) identified from soybeans in Korea, with 99% coverage and 99.27% nucleotide identity.

### 2.2. Examination of 143 Complete SMV Genomes Based on Country, Plant Host, and Year of Identification

**Figure 1:**
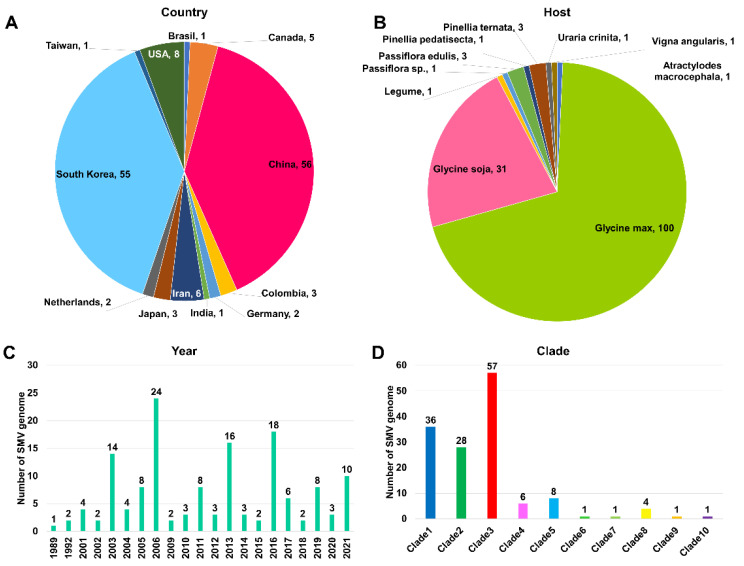
Information for 143 SMV genomes and the 10 identified clades. Pie charts display information for each country where SMV genomes were obtained (**A**) and the host plants from which SMV genomes were obtained (**B**). The bar graph shows the number of SMV genomes based on the year of identification (**C**). Information for the 10 clades of 143 SMV genomes that were identified using phylogenetic analysis (**D**).

We downloaded all SMV sequences from GenBank and only used SMV genomes with genome sequences that were over 9000 nt in length, meaning that the whole coding DNA sequence was covered. The selection process resulted in 136 SMV genome sequences from GenBank. We also included 7 SMV genome sequences from this study, which resulted in the analysis of a total of 143 SMV genome sequences. First, we examined the distribution of 143 SMV genomes by country (Figure 1A). The SMV genomes we studied were obtained from 12 countries: Brazil, Canada, China, Colombia, Germany, India, Iran, Japan, the Netherlands, Taiwan, and the USA. The majority of the SMV genomes were derived from two countries, China (56 genomes) and South Korea (55 genomes), followed by the USA (eight genomes) and Canada (five genomes). Next, we examined the distribution of the SMV genomes in plant hosts. The SMV genomes were identified in ten plant species: *Atractylodes macrocephala*, *Glycine max*, *Glycine soja*, other legumes, *Passiflora* species (including *Passiflora edulis*), *Pinellia pedatisecta*, *Pinellia ternata*, *Uraria crinita*, and *Vigna angularis* (Figure 1B). Of these, two plant species, *G. max* (100 genomes) and *G. soja* (31 genomes), were identified as major hosts for SMV.

Since the first SMV genome was reported in 1989, many SMV genomes have been sequenced (Figure 1C). Before 2000, only three complete genome sequences of three SMVs had been reported. However, the rapid development of sequencing technologies, such as HTS, has dramatically increased the number of complete SMV genome sequences. In 2006 and 2016, many SMV genomes were reported (24 and 18 genomes, respectively).

### 2.3. Phylogenetic Analysis of 143 SMV Genomes

**Figure 2:**
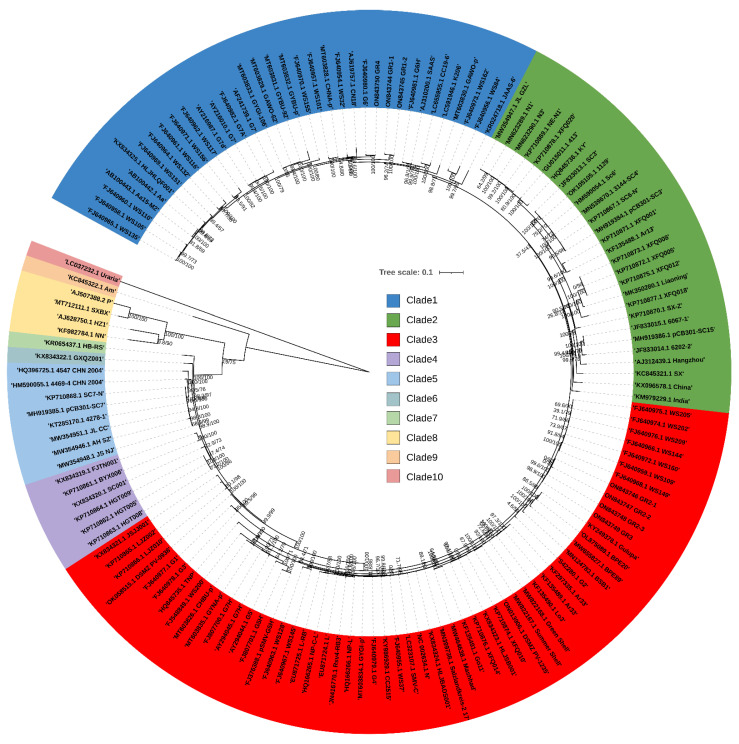
Phylogenetic tree of 143 SMV genomes constructed based on complete coding DNA sequences, generated using the maximum likelihood method. A total of 143 sequences with 9805 sites covering whole coding DNA sequences were used for phylogenetic tree construction using IQ-TREE with GTR + F+I + G4 for the best-fit model. The accession number and isolate (strain) name for each SMV genome are indicated. Both SH-aLRT and UFBoot support values are indicated on the branches. A total of 10 clades were found, and they are shown in 10 different colors. The isolate Uraria (GenBank LC037232.1) in clade 10 was used as an outgroup. Detailed information for individual SMV genomes can be found in Table S1.

**Figure 3:**
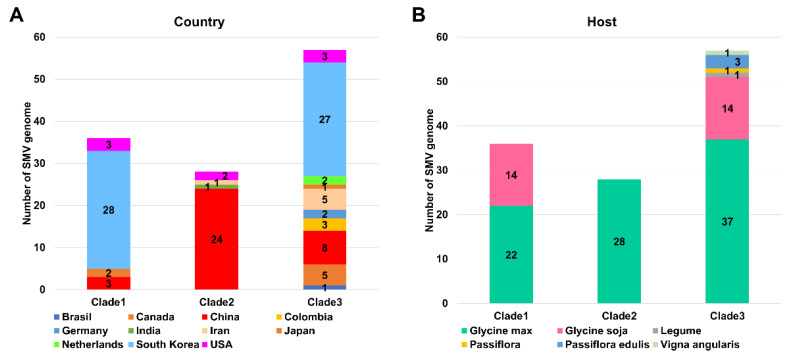
Composition of SMV isolates in the three major clades that became apparent when the isolates were analyzed based on their country of origin and host plant. The number of SMV isolates in each of the three major clades (clades 1 through 3) based on country of origin (**A**) and host plant (**B**) are indicated.

We constructed a maximum likelihood (ML) phylogenetic tree using the 143 SMV coding DNA sequences (CDS) that we selected (Figure 2). We identified ten clades based on the ML phylogenetic tree (Figure 2). Three clades (clades 1, 2, and 3) were identified as major clades, and three (clades 8, 9, and 10) were identified as minor clades (Figure 1D). The SMV genomes in clade 1 were mostly derived from South Korea (28 genomes), whereas the SMV genomes in clade 2 were mostly derived from China (24 genomes) (Figure 3A). In clade 3, the majority of SMV genomes were derived from South Korea (27 genomes), followed by China (eight genomes), and Japan (five genomes). In clades 1 and 2, the SMV genomes were derived from four countries, whereas the SMV genomes in clade 3 were derived from ten countries. When the genomes were analyzed based on their identified plant hosts, the SMV genomes in clade 1 were identified as coming from *G. max* (22 genomes) and *G. soja* (14 genomes), whereas all the SMV genomes in clade 2 were derived from *G. max* (28 genomes) (Figure 3B). In clade 3, the SMV genomes came from six plant species. Among these, *G. max* (37 genomes) was the dominant plant host, followed by *G. soja* (14 genomes). The phylogenetic tree showed that most of the SMV genomes were closely related (Figure 2). However, 6 SMV genomes in clades 8, 9, and 10 were identified in China and were phylogenetically distant from the other 137 SMV genomes. The four SMV genomes in clade 8 were derived from *P. ternata* (three genomes) and *P. pedatisecta* (one genome), whereas the Am isolate in clade 9 and the Uraria isolate in clade 10 were found in *A. macrocephala* and *U. crinita*, respectively. During data analysis, we found that the SMV isolate Uraria (LC037232.1) from *Uraria crinita* in clade 10 showed strong sequence similarity to uraria mosaic virus (UMV) isolate OC (LC477217.1) [17]. Based on the molecular species demarcation for potyviruses, the SMV isolate Uraria should be revised to UMV isolate Uraria. Our results showed that 142 SMV isolates/strains in this study could be classified into 9 clades, and that the UMV isolate Uraria in this clade 10 could be used as an outgroup.

### 2.4. Recombination Analysis of 143 SMV Isolates/Strains

**Figure 4:**
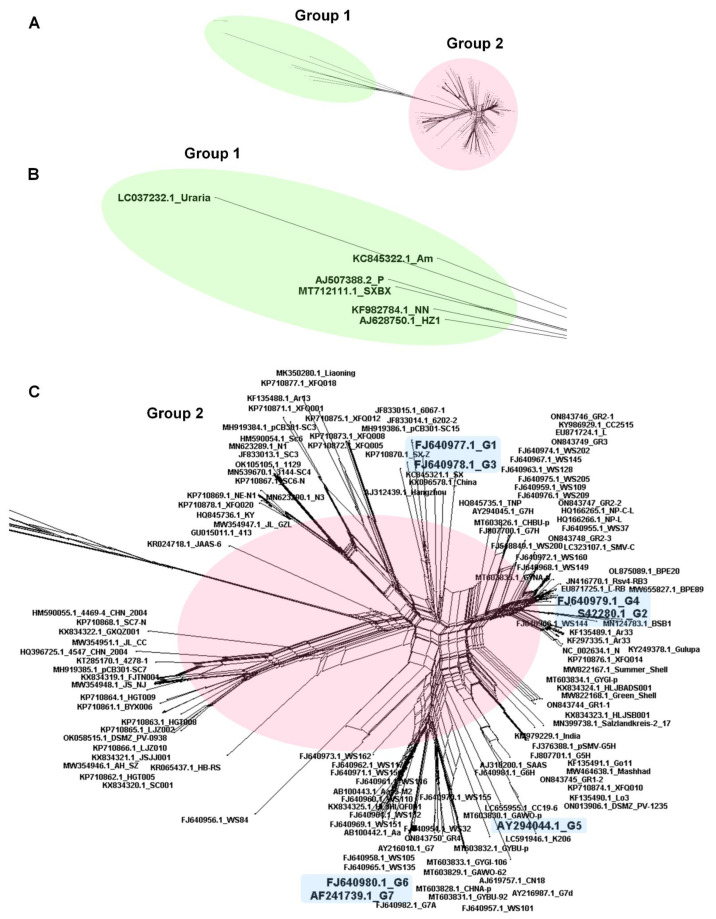
Phylogenetic network tree of 143 SMV genomes. The network tree was rooted using the complete genome sequences of 143 SMV genomes and contains 2 groups (Group 1 and Group 2) of SMV genomes (**A**). Group 1 is shown in a green oval in the network tree and was then further magnified (**B**). Group 2 is shown in a pink oval in the network and then was further magnified (**C**) The seven known SMV strains (G1–G7) are shown in the light blue colored box. The accession numbers and isolate names are also indicated. Using the nexus alignment file in the supplemental file S1, the network tree can be visualized in the SplitsTree program.

Recombination is an important process contributing to the genetic diversity of RNA viruses. To examine the recombination events in the 143 SMV isolates, all complete genome sequences were aligned and subjected to recombination analysis. First, we performed a network analysis using the SplitsTree4 program to examine the possible presence of recombinants in the 143 SMV genomes. The network tree showed many reticulated nodes, indicating that a high frequency of genetic recombination occurred among different SMV isolates/strains (Figure 4A). It was difficult to distinguish between several groups based on the network tree. However, the network tree revealed two major groups of SMV isolates/strains, designated as group 1 and group 2 (Figure 4B,C). Furthermore, we examined whether the seven known SMV strains G1–G7 clustered in different reticulated nodes (Figure 4C). We found that SMV strains G1 and G3 grouped together, and that SMV strains G2 and G4 also grouped together.

**Figure 5:**
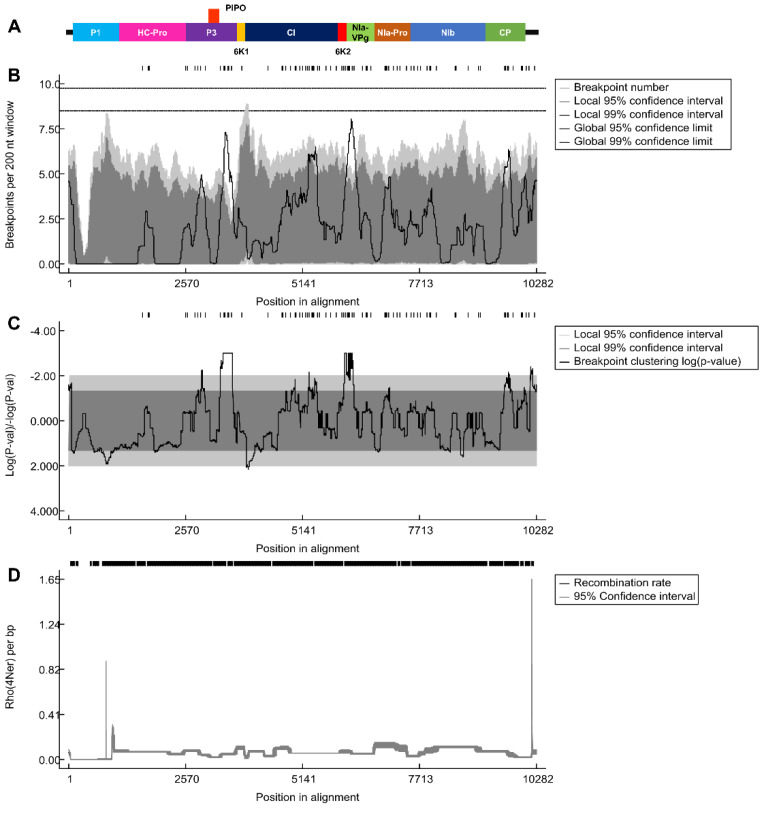
Recombination breakpoint distribution plots for the SMV genome. SMV genome organization showing individual protein regions (**A**). Recombination breakpoint distribution plot (**B**) and respective statistical log converted *p*-value (**C**). Locally and globally significant breakpoint clusters were identified using 95% and 99% permutation tests. Recombination rate plot (**D**).

The aligned 143 SMV genome sequences were subjected to recombinant analysis using the RDP program (Table S2). A total of 76 recombinant events were identified. Of the 143 SMV isolates, 141 were predicted to be recombinants. The two non-recombinant SMV isolates were SMV isolate WS84 (FJ640956.1) from *G. soya* in Korea and SMV isolate Uraria (LC037232.1) from *Uraria crinita* in Taiwan, which were used as outgroups in the phylogenetic tree construction. Next, we determined whether the recombinant breakpoints were uniformly distributed across the SMV genome (Figure 5A). For this purpose, we calculated the breakpoints using a sliding window of 200 nt (Figure 5B). Most regions of the SMV genome showed a high number of breakpoints with strong statistical support (Figure 5C). For example, P1 had a relatively intact region, referred to as a cold spot, followed by P3. The regions with the highest number of breakpoints, referred to as hot spots, were found between the 6K1 and CI regions and between P1 and NIa-Pro (Figure 5B). In contrast, the recombination rate plot indicated by rho (4Ner) showed the highest value at the end of the 3′ region of the SMV genome, followed by the P1 region (Figure 5D). Except for these two regions, we did not observe any significant changes in the recombination rate across the SMV genome.

**Figure 6:**
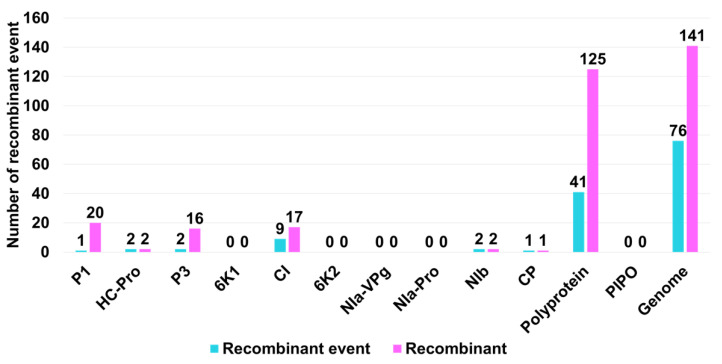
Number of recombinant events and recombinants in individual SMV proteins or genomes.

Next, we tested whether recombination occurred frequently within the individual protein sequences. To this end, we conducted recombination analyses using nucleotide sequences corresponding to the individual proteins. We identified 41 recombination events and 125 recombinants in the polyprotein sequences, whereas no recombinant events were identified in PIPO (Figure 6). Among the mature proteins processed from the polyprotein, CI displayed the highest number of recombination events (9 events) and recombinants (17 recombinants). P1 and P3 had one and two recombinant events, respectively. However, the number of recombinants identified for P1 (20) and P3 (16) was very high. We identified two recombinant events and recombinants in both HC-Pro and NIb, whereas a single recombinant and recombination event was found in CP.

No recombinant events were found in five SMV proteins, namely, 6K1, 6K2, Nia-VPg, Nia-Pro, and PIPO. Furthermore, we examined the association between breakpoint distribution and specific genomic regions (protein-coding regions). Three protein regions, namely, CI, 6K2, and PIPO, showed high breakpoint levels compared to those of the other SMV genome regions.

### 2.5. Genetic Diversity Analysis of 143 SMV Genomes

We also analyzed the genetic diversity of 143 SMV genomes, polyproteins, PIPOs, and 10 mature proteins processed from SMV polyproteins. The genetic diversity of the complete genome sequence was S = 8653 (number of segregating sites), Eta = 25946 (total number of mutations), Pi = 0.62182 ± 0.01438 (nucleotide diversity), *θw* = 0.18062 ± 0.04072 (Watterson’s estimator of θ), and Tajima’s D = 0.49152 (Table 1). The genetic diversity of the polyprotein was S = 4375, Eta = 6181, Pi = 0.06895 ± 0.00516, *θw* = 0.09303 ± 0.02100, and Tajima’s D = −1.57646, and the genetic diversity of PIPO was S = 92, Eta = 106, Pi = 0.02977 ± 0.00394, *θw* = 0.07418 ± 0.01833, and Tajima’s D = −2.07972. Of the ten mature proteins processed from the polyprotein, CI (Eta = 1335) had the highest number of mutations, whereas 6K2 had the lowest number of mutations. Haplotype analysis revealed that most SMV proteins exhibit a high level of haplotype diversity. CI (number of haplotypes, H = 131) showed the highest haplotype diversity (Hd = 0.9986 ± 0.0011), whereas 6K2 (H = 66) displayed the lowest haplotype diversity (Hd = 0.957 ± 0.011). According to the Pi value for nucleotide diversity, nucleotide diversity was the highest in P1 (Pi = 0.11369 ± 0.01319) and lowest in PIPO (Pi = 0.02977 ± 0.00394).

Next, we calculated the average number of segregated sites (*θw*) for each protein. Again, P1 (*θw* = 0.14546 ± 0.03353) showed the highest value of *θw*, followed by 6K1 (*θw* = 0.10768 ± 0.02658) and P3 (*θw* = 0.10100 ± 0.02311). PIPO (*θw* = 0.07418 ± 0.01833) had the lowest *θw*. To test the neutral mutation hypothesis, we calculated Tajima’s D for individual SMV proteins [18]. All the 11 mature SMV proteins had negative Tajima’s D values, ranging from −2.07972 (PIPO) to −1.38761 (HC-Pro). Only three proteins with small sizes (6K1, 6K2, and PIPO) showed statistically significant differences (*p* < 0.05) for Tajima’s D values. Tajima’s D value for the SMV genome was positive (0.49152), but this observation was not statistically supported (*p* > 0.10).

**Figure 7:**
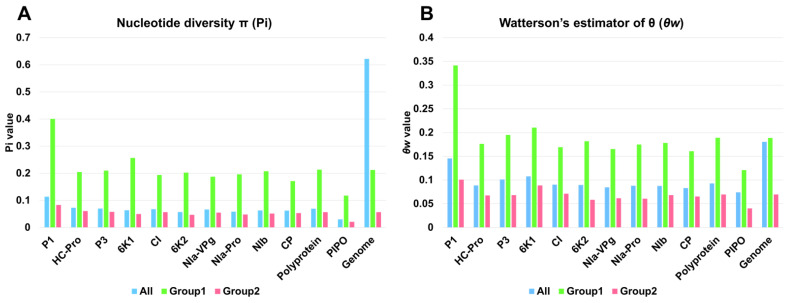
Genetic diversity of SMV proteins or genomes in different groups of SMV. Nucleotide diversity (Pi) (**A**) and average number of segregating sites (*θw*) (**B**) of individual SMV proteins or genomes in the three groups. ‘All’ indicates all 143 SMV genomes, and group 1 and group 2 include 6 and 137 SMV genomes, respectively.

Next, we compared the nucleotide diversity (Pi) and the average number of segregating sites (*θw*) in the two SMV groups that were defined using network analysis (Tables S3 and S4). Group 1 contained 6 SMV genomes, whereas group 2 contained 137 SMV genomes. The nucleotide diversity values of all the SMV proteins in group 1 were always higher than those of group 2 and those of all 143 SMV genomes indicated by ‘all’ (Figure 7A). The nucleotide diversity for all 143 SMV genomes was 0.62182, which was much higher than that for group 1 (Pi = 0.21194) and group 2 (Pi = 0.056569). Among the SMV proteins, P1 (Pi = 0.40057) showed the highest nucleotide diversity in group 1, whereas PIPO (Pi = 0.02064) showed the lowest nucleotide diversity in group 2.

The average number of segregating sites (*θw*) for all SMV proteins in group 1 was much higher than that in group 2 (Figure 7B). However, the difference in the average number of segregating sites between ‘all’ (Pi = 0.18062) and group 1 (Pi = 0.18856) was small. The average number of segregating sites for P1 (*θw* = 0.14546) in group 1 was the highest among the SMV proteins, whereas PIPO (*θw* = 0.0401) in group 2 had the lowest average number of segregating sites.

### 2.6. Estimation of the Nonsynonymous to Synonymous Rate Ratio of all SMV Protein Sequences

The ratio of nonsynonymous to synonymous substitutions (dN/dS), also known as Ka/Ks, is a useful measure of natural selection in protein-coding genes [19]. The average dN/dS ratio was greater than one for the SMV genome (1.004237), polyprotein (1.568258), and P1 (1.066121), whereas the average dN/dS ratio for the other ten mature SMV proteins was less than one (Table 2). The maximum dN/dS ratio was the highest for the polyprotein, followed by P1. In contrast, the maximum dN/dS ratio for NIa-VPg was the lowest among all examined SMV proteins. The number of nonsynonymous sites was much higher than that of synonymous sites for all examined proteins, except 6K2, in which the number of synonymous sites was approximately 1.2 times higher than that of nonsynonymous sites.

**Figure 8:**
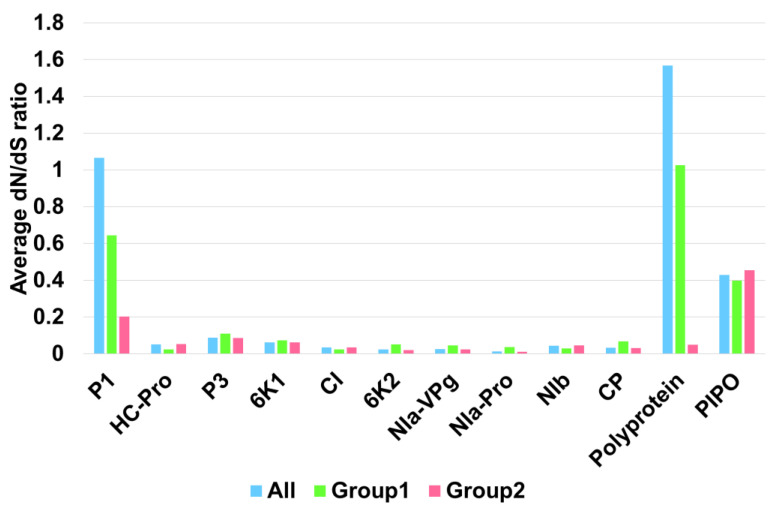
Average dN/dS ratio of SMV proteins in three groups. ‘All’ indicates the 143 SMV genomes, whereas group 1 and group 2 include 6 and 137 SMV genomes, respectively.

Next, we compared the average dN/dS ratio for all SMV proteins in three different groups, ‘all’, group 1, and group 2 (Table S5 and Figure 8). The average dN/dS ratios for the polyprotein and P1, followed by PIPO, were much higher than those for the other SMV proteins. The average dN/dS ratios for most SMV proteins were less than one except for P1 and polyprotein in ‘all’ and group 1. The average dN/dS ratios for P1 in ‘all’ and group 1 were 1.06612 and 0.64502, respectively. The average dN/dS ratios for polyprotein in ‘all’ and group 1 were 1.56825 and 1.02581, respectively. The average dN/dS ratios for PIPO in all three groups were comparable, ranging from 0.45433 (group 2) to 0.39788 (group 1). The lowest average dN/dS ratio was 0.01168 for NIa-Pro in group 2. The highest average dN/dS ratio was 1.56825 for polyprotein in group 1. Most SMV proteins, such as HC-Pro, P3, 6K1, CI, 6K2, NIa-VPg, NIa-Pro, Nib, and CP, had average dN/dS ratios of less than 0.2. 

## 3. Discussion

In this study, we found seven complete SMV genome sequences from four soybean germplasms using RNA sequencing. Of the four soybean germplasms, two germplasms, GR1 and GR2, contained two and three SMV isolates, respectively. This result indicated that a single soybean seed can contain more than one SMV variant at a time. The nucleotide identity between the SMV isolates in GR1 was 99.68%, whereas that among the three SMV isolates in GR2 ranged from 99.87% to 99.26%. This result indicates that the SMV isolates within the same soybean seed have a high degree of sequence similarity. In RNA viruses, a viral quasispecies is defined as a viral population structure consisting of several viral variants (isolates) that play an important role in maintaining the genetic diversity of RNA viruses [20]. Seed transmission of SMV is well known; however, the seed transmission rate for SMV may vary [21]. Our previous study showed a high frequency of SMV mutations in soybean seed transcriptome data [22]. Thus, we showed that viral quasispecies contribute to SMV diversity within the soybean germplasm.

In RNA viruses, recombination, i.e., the exchange of genetic segments between two viral genomes, plays an important role in viral diversity and evolution [23]. As expected, the 143 SMV genomes showed a high number of recombinant events (76), and most SMV genomes (141) were predicted to be recombinants. Several previous studies have also revealed recombination events among SMV isolates/strains. Recombination events have also been identified between distinct SMV pathotypes [24]. A previous study revealed 19 SMV recombinant events in 44 SMV isolates, of which 30 were derived from Korea [12]. Another study revealed 32 recombination events in 83 SMV isolates, including 18 newly sequenced Chinese SMV isolates [13]. As the number of SMV genomes used for recombination analysis increases, the number of recombination events also seems to increase. Interestingly, a previous study suggested that two SMV isolates, 4547_CHN_2004 (HQ396725) and 4469-4_CHN_2004 (HM590055.1), could be derived from a recombination event between SMV and bean common mosaic virus (BCMV), and they were referred to as the SMV-R (recombination) strain [25]. Based on this result, we suggest that some SMV isolates in clade 5 containing two SMV-R strains might be recombinants between SMV and BCMV. Thus, the effect of recombination on the genetic diversity of SMV genomes may be much greater than expected. Moreover, we found no specific protein regions in which recombination occurred frequently. Recombination events occurred in most SMV genome regions covering the polyprotein, except for the P1 region, which was identified as a cold spot for recombination. 

A previous study using 83 SMV genome sequences identified 4 major clades [13]. Phylogenetic analysis using SMV complete coding sequences revealed 9 clades that collectively contained 142 SMV isolates/strains. It is possible that a phylogenetic tree with a higher number of viral genome sequences has a higher number of clades. 

Of the nine clades, clades 1, 2, and 3 contained a higher number of SMV isolates/strains than the other clades. We identified seven previously well-defined SMV strains (G1 through G7) based on pathotypes belonging to clade 1 (G6 and G7) and clade 3 (G1 through G5) [26]. SMV isolates in clades 1 and 3 were identified in diverse countries, and South Korea was a major source of these isolates. Moreover, SMV isolates in clade 1 and clade 3 were found in diverse plant species, including *G. max* and *G. soja*. These results suggest that SMV isolates in clades 1 and 3 are the most common SMV pathotypes, with a worldwide distribution. In particular, they have been found in South Korea and the USA. In contrast, SMV isolates in clade 2, derived mostly from *G. max* in China, were unique compared to those in the other clades. Two types of SMV strains have been identified in China. The first Chinese SMV type consists of 22 SMV SC strains isolated from soybean cultivars in southern China [27]. The second SMV Chinese type contains the N1–N3 strains, which were identified by evaluating soybean cultivars from northeast China [28]. Clade 2 contained the N1, N3, SC3, SC6, and SC6-N strains, which were previously identified as SMV strains from China. Therefore, the SMV isolates in clade 2 may be related to the well-known Chinese SMV pathotypes that originated in and are now distributed throughout China. In addition, 16 SMV isolates in clades 4 through 7 were derived from China and showed high sequence diversity when compared with other SMV isolates identified in *G. max* and *G. soja*. This result indicates that the wide geographical distribution of soybean cultivation in China may contribute to the genetic diversity of the SMV isolates. The most unique clades of the SMV isolates were clades 8 and 9. Clade 8 consists of four SMV isolates from two *Pinellia* species, whereas clade 9 contains a single SMV isolate from *Atractylodes macrocephala*. These results suggest that plant hosts are a powerful force driving mutations in the SMV genome.

Nucleotide diversity and Watterson estimator results for the nucleotide sequences corresponding to individual SMV proteins showed values that were all lower than one, indicating that all the SMV gene sequences analyzed were under negative selection. However, nucleotide diversity in the P1 region was much higher than that in other SMV protein regions, suggesting a high degree of nucleotide diversity in the P1 region. A comparison of the two groups of SMV isolates based on their plant hosts showed that the nucleotide diversity of SMV isolates in group 1, which were derived from *Pinellia* and *Atractylodes* species, was much higher than those in group 2, which were derived mostly from *G. max* and *G. soja*. The dN/dS ratio results for natural selection pressure revealed that most SMV protein sequences were negatively selected, except for P1, which showed positive selection in all analyzed SMV sequences. Our results are consistent with those of a recent study that used 104 complete SMV sequences [29]. However, the dN/dS ratios for P1 within group 1 and group 2 were less than one, indicating negative selection. Thus, we found that the P1 region of SMV isolates in group 1 contributes to the positive selection of the P1 protein. Moreover, a recent study based on the comparison of two different SMV isolates with high sequence variability in the P1 region suggested that the P1 protein of SMV might be related to viral adaptation to different hosts [30]. Both our results and that of previous studies suggest that positive selection and high sequence variability in the P1 protein of SMV isolates in group 1 might be highly correlated with host adaptation abilities.

The SMV polyprotein sequences in ‘all’ and group 1 showed positive selection. Again, the dN/dS ratio (1.568) for the polyprotein using all SMV sequences was significantly reduced in group 1 (1.025) after SMV isolates in group 1 were removed. Furthermore, the dN/dS ratio for the polyprotein was higher than that for the PIPO. Interestingly, the dN/dS ratios for PIPO in the three different groups were quite similar, suggesting the genetic conservation of PIPO sequences in strains derived from different plant species. Taken together, our results provide strong evidence that plant hosts such as *Pinellia* and *Atractylodes* species play important roles in maintaining the genetic diversity of the SMV genome.

## 4. Materials and Methods

### 4.1. Soybean Germplasms

Germplasms are defined as living genetic resources maintained as seeds and plant tissues. Before identifying soybean genetic resources resistant to SMV, we used RT-PCR to examine viral infections in 150 different soybean germplasms derived from the National Agrobiodiversity Center of the Rural Development Administration. In June 2020, soybean seeds, which were representative of the soybean germplasm, were planted in plastic nursery pots in a greenhouse at the National Institute of Crop Science in Wanju, Korea.

### 4.2. Total RNA Extraction and Library Zpreparation

We harvested mature trifoliate leaves from soybean seedlings and extracted total RNA using an RNeasy Plant Mini Kit (Qiagen, Hilden, Germany), according to the manufacturer’s instructions. The extracted total RNA was used for reverse transcriptase-polymerase chain reaction (RT-PCR) analysis using SMV-specific primers. We selected 10 soybean germplasms that were infected with SMV. The quality of the extracted total RNAs was measured using a 2100 Bioanalyzer (Agilent Technologies, Santa Clara, CA, USA). For library preparation, we used total RNAs with an RNA integrity number (RIN) value greater than or equal to seven. After removing ribosomal RNA from the total RNA using TruSeq Stranded Total RNA with a Ribo-Zero Plant Kit (Illumina, San Diego, CA, USA), we generated 10 libraries for RNA sequencing using the TruSeq Stranded Total RNA LT Sample Prep Kit (Illumina).

### 4.3. RNA-Sequencing and Viral Genome Assembly

Ten libraries representing ten soybean germplasms were paired-end (2 × 101 bp) sequenced using a NovaSeq 6000 system (Macrogen, Seoul, Republic of Korea). We performed de novo transcriptome assembly using the raw sequence reads obtained from the Trinity assembler (version 2.13.2) with default parameters. The assembled contigs in each transcriptome were used for a BLASTX search against the viral reference protein database (21 August 2022) obtained from the National Center for Biotechnology Information (NCBI) using the DIAMOND program with an E-value of 1 × 10^−5^ as the cutoff [31]. Using the BLASTX results, we selected only virus-associated contigs. All virus-associated contigs were derived from SMV. Of the SMV-associated contigs, we selected viral contigs with sizes greater than 9000 bp to identify SMV sequences that covered all open reading frames (ORFs) using the NCBI ORFfinder [32].

### 4.4. Collection of SMV Genomes from GenBank

Using SMV as a query, we searched for and downloaded all SMV-associated sequences from GenBank in the NCBI database (11 August 2022). We obtained 667 SMV sequences from the GenBank database. In this study, we combined 667 sequences with 7 SMV genome sequences, resulting in a total of 674 sequences. Using reformat.sh from the BBMap suite, we deleted sequences of less than 9000 bp. After deleting redundant sequences, we obtained 143 SMV genome sequences.

### 4.5. Generation of Phylogenetic Trees

A total of 143 SMV genome sequences were aligned using MAFFT version 7 with the L-INS-i option [33]. Subsequently, we extracted only coding DNA sequences (CDSs) for 143 SMV genomes by deleting the 5′ and 3′ untranslated regions using the MEGA7 program [34]. After the alignment of the 143 coding DNA sequences, they were subjected to phylogenetic tree construction using IQ-Tree version 2.2.0 [35]. We used ModelFinder implemented in IQ-Tree for fast model selection [36]. Then, 2 fast approximate likelihood-based measures of branch support, SH-aLRT and ultrafast bootstrap, were conducted with 1000 bootstrap replicates. The generated maximum likelihood phylogenetic tree was imported into Figtree version 1.4.4 (http://tree.bio.ed.ac.uk/software/figtree/, (accessed on 1 August 2022)). The phylogenetic tree was modified using iTOL version 4 [37].

### 4.6. Recombination Analysis and Network Tree for 143 SMV Genomes

We conducted recombination analyses for 143 SMV genomes using RDP version 5.23 [38]. A full exploratory recombination scan using all seven available detection methods, namely RDP, GENECONV, Bootscan, Maxchi, Chimaera, SiSscan, and 3Seq, was used for the recombination analyses. Of the seven algorithms, recombinants predicted by at least five algorithms with a *p*-value less than 0.05 were regarded as true recombinants. We rechecked the identified recombinant events using all seven detection methods. An unrooted network tree based on the aligned 143 SMV genome sequences was generated using SplitsTree version 5.3.0 [38].

### 4.7. Analyses of Genetic Diversity and Selection Pressure for Individual SMV Proteins

After aligning the 143 SMV genome sequences, we manually extracted nucleotide sequences corresponding to individual SMV proteins. For example, the sequences of the two ORFs encoding polyproteins and PIPO were extracted first. Next, we obtained nucleotide sequences corresponding to the ten mature proteins cleaved from the SMV polyprotein. As a result, the nucleotide sequences of 12 SMV proteins were used for genetic diversity analysis using DnaSP version 6.12.03 [39]. The genetic diversity indices included the total number of sites, number of segregating sites (S), total number of mutations (Eta), number of haplotypes (H), haplotype diversity (Hd), nucleotide diversity (Pi), and Watterson’s estimator of θ (θw).

We calculated the dN/dS ratio for the 12 individual SMV proteins to determine selection pressure using DnaSP. dN represents the average number of nonsynonymous substitutions per non-synonymous site, whereas dS represents the average number of synonymous substitutions per synonymous site. A dN/dS ratio of less than one indicates negative selection (purifying), whereas a dN/dS ratio greater than one indicates positive selection (diversifying). A dN/dS ratio of zero indicates neutral selection. 

## 5. Conclusions

In this study, we obtained seven SMV genome sequences from soybean germplasms using RNA sequencing and combined all available SMV genome sequences, totaling 143 SMV genomes. Then we conducted a large-scale comprehensive evolutionary and phylogenetic study of these SMV genomes, which were identified from 10 plant species and 12 countries. A maximum likelihood phylogenetic tree constructed using the polyprotein sequences showed that the 142 SMV isolates/strains grouped into 9 clades. A recombination analysis revealed 76 recombinant events and 141 recombinants. Most regions of the SMV genome showed a high number of breakpoints, whereas P1 was identified as a cold spot. Clades 1 and 3 contain the most common SMV pathotypes, including G1 through G7, which are distributed worldwide, whereas clade 2 contains SMV pathotypes that are distributed in China and might have originated there. The SMV isolates were further divided into two groups. The SMV isolates in the first group were identified in *Pinellia* and *Atractylodes* species, whereas the second group was identified mostly in cultivated soybeans. The SMV polyprotein undergoes positive selection, whereas most mature proteins undergo negative selection. The only exception we found was the P1 protein. The P1 protein of SMV isolates in group 1 may be highly correlated with host adaptation. Taken together, our results provide strong evidence that recombination and plant hosts are powerful forces that drive mutations and genetic diversity in the SMV genome.

## Figures and Tables

**Table 1:** Genetic diversity of 143 SMV genomes based on nucleotide sequences corresponding to individual proteins.

Protein	Sites	S	Eta	H	Hd	Pi	*θw*	Tajima’s *D*
P1	1436	393	641	99	0.98 ± 0.003	0.11 ± 0.01	0.14 ± 0.03	−1.71
HC-Pro	1374	668	948	124	0.99 ± 0.001	0.07 ± 0.005	0.08 ± 0.02	−1.38
P3	1041	581	808	126	0.99 ± 0.001	0.06 ± 0.005	0.1 ± 0.02	−1.65
6K1	156	93	137	76	0.97 ± 0.005	0.06 ± 0.006	0.1 ± 0.02	−1.92
CI	1902	949	1335	131	0.99 ± 0.001	0.06 ± 0.004	0.09 ± 0.02	−1.55
6K2	159	79	114	66	0.95 ± 0.01	0.05 ± 0.004	0.08 ± 0.02	−1.78
NIa-VPg	576	264	360	103	0.99 ± 0.002	0.06 ± 0.004	0.08 ± 0.01	−1.38
NIa-Pro	729	335	461	112	0.99 ± 0.001	0.05 ± 0.004	0.08 ± 0.02	−1.70
NIb	1551	648	894	125	0.99 ± 0.001	0.06 ± 0.004	0.08 ± 0.02	−1.58
CP	873	365	483	118	0.99 ± 0.001	0.06 ± 0.004	0.08 ± 0.01	−1.41
Polyprotein	9803	4375	6181	142	0.99 ± 0.0008	0.06 ± 0.005	0.09 ± 0.02	−1.57
PIPO	225	92	106	72	0.96 ± 0.01	0.02 ± 0.003	0.07 ± 0.01	−2.07
Genome	10,044	8653	25,946	143	1.000 ± 0.0008	0.62 ± 0.01	0.18 ± 0.04	0.49

Sites: total number of sites; S: number of segregating sites; Eta: total number of mutations; H: number of haplotypes; Hd: haplotype diversity; Pi: nucleotide diversity; *θw*: Watterson’s estimator of θ.

**Table 2:** Summary of synonymous site and nonsynonymous sites for individual SMV protein sequences.

Protein	Number of Sites	Average	Min	Max	Synonymous Site	Nonsynonymous Sites
P1	1436	1.066121	0	6.822368	99.16	356.84
HC-Pro	1374	0.050681	0	1.117647	294.73	1064.28
P3	1041	0.08778	0	1.326087	217.4	817.6
6K1	156	0.062452	0	1.373193	30.8	125.2
CI	1902	0.0342	0	1.173913	431.7	1467.3
6K2	159	0.023262	0	0.754717	32.24	26.76
Nia-VPg	576	0.025513	0	0.560976	121.22	439.78
Nia-Pro	729	0.012687	0	0.823529	147.79	536.21
Nib	1551	0.04367	0	2.2	288.16	1046.84
CP	873	0.0326	0	0.830508	169.69	619.31
Polyprotein	9803	1.568258	0	10	1702.53	6721.47
PIPO	225	0.429524	0	4.219048	47.26	174.74
Genome	10,044	1.004237	0	3.685714	1776.3	6854.7

Average, Min, and Max indicate the average, minimum, and maximum ratios of dN/dS, respectively, for the given protein sequences.

## Data Availability

The seven SMV genome sequences used in this study are publicly available in the NCBI GenBank database with the following accession numbers: ON843744–ON843750.

## Supplementary Materials

The supporting information can be downloaded at: https://www.mdpi.com/article/10.3390/ijms24010022/s1.
